# The Impact of Societal Ageing on Individual Consumers’ Insurance Purchase Intentions: A Review and Research Agenda

**DOI:** 10.3390/bs16010143

**Published:** 2026-01-20

**Authors:** Mohd Hafizuddin-Syah Bangaan Abdullah, Zhangwei Zheng, Hafizah Omar Zaki, Qin Lingda Tan

**Affiliations:** Faculty of Economics and Management, Universiti Kebangsaan Malaysia, Bangi 43600, Selangor, Malaysia; m_hafiz@ukm.edu.my (M.H.-S.B.A.); hafizah.omar@ukm.edu.my (H.O.Z.); p132640@siswa.ukm.edu.my (Q.L.T.)

**Keywords:** ageing, insurance purchase intention, age groups, systematic review, TCCM framework, risk perception

## Abstract

This study examines how societal ageing influences insurance purchasing intentions, addressing the prevailing emphasis on elderly consumers and the limited conceptual integration of ageing within existing behavioural models. Following the Preferred Reporting Items for Systematic Reviews and Meta-Analyses (PRISMA) guidelines, a systematic review was conducted using an initial search (forty-three studies) supplemented by a top-up search in November 2025 that identified fourteen additional articles. Using the theories-contexts-characteristics-methods (TCCM) framework, the review synthesises theoretical, contextual, characteristic, and methodological patterns in this field. The findings indicate that although variables such as risk perception, anticipated dependence, and interpersonal influence are frequently examined, ageing itself is seldom conceptualised as an explanatory construct, constraining theoretical precision and practical relevance. To bridge this gap, the study introduces ageing risks (AR)—capturing perceived financial, health, and intergenerational uncertainties associated with demographic ageing—and illustrates its integration within the Theory of Planned Behaviour (TPB). The review highlights the need to validate AR empirically, extend research to non-elderly populations and underexplored regions, and broaden methodological approaches. These contributions strengthen theoretical development and inform more responsive insurance strategies in ageing societies.

## 1. Introduction

The phenomenon of ageing has become a focal point of global scholarly attention. Defined by a rising proportion of older individuals and unprecedented increases in life expectancy, ageing is reshaping demographic structures and driving complex societal changes (Cox, 1956; Zheng et al., 2025a). This demographic shift extends beyond population composition, influencing healthcare systems (Foster, 2018), pension schemes (Jaafar et al., 2019), and social support frameworks (Nagarajan & Sixsmith, 2023). While ageing is commonly associated with the elderly, it is increasingly recognised as a broader societal phenomenon that affects individuals across all age groups, reshaping family dynamics (Patterson, 2007), workforce structures (Adnand et al., 2021), and individual decision-making (Zheng et al., 2024b).

Despite extensive research on ageing and its societal implications, its influence on insurance purchasing intentions remains underexplored (Zheng et al., 2024a, 2025a). Studies have primarily examined demographic or economic determinants of insurance demand (Outreville, 1996; D. Li et al., 2007), household insurance behaviour (Y. Lin & Grace, 2007; Frees & Sun, 2010), and macro-level impacts of ageing on financial systems (Baharin & Saad, 2018; Nebolsina, 2020; G. Li et al., 2021). Existing research often focuses on older individuals, analysing how caregiving experiences shape insurance considerations (Alavi et al., 2010; Tennyson & Yang, 2014; Broyles et al., 2016), how expectations of dependency influence planning (Costa-Font & Courbage, 2015; Xu et al., 2020; He et al., 2023), and how risk perceptions affect insurance decisions (Tolani et al., 2019; Choe et al., 2022; Wu & Gong, 2023). However, research rarely considers the broader impact of ageing as a social construct, particularly on non-elderly individuals, who may also adjust their insurance decisions in response to ageing-related societal trends. For example, families with both elderly and younger dependents may prioritise insurance differently from those without caregiving responsibilities (Tennyson & Yang, 2014; Broyles et al., 2016). Similarly, non-elderly consumers with chronic illnesses may be more attuned to the financial risks of ageing (Guillemette et al., 2016; Tam et al., 2021).

From a practical perspective, the evolution of the insurance industry necessitates a nuanced understanding of consumer behaviour (Al Mamun et al., 2022; Bangaan Abdullah et al., 2022). As ageing becomes a global concern, understanding its influence on insurance purchasing intentions is essential for addressing diverse consumer needs and enhancing insurance penetration worldwide. Such insights can guide policymakers, insurers, and brokers in fostering sustainable growth in the sector. Against this backdrop, a systematic review of existing research is both timely and necessary to provide actionable insights for academics and practitioners alike (Zheng et al., 2024a).

A review of existing literature reviews reveals that limited attention has been given to the relationship between ageing and insurance purchasing intentions. While Zniva and Weitzl (2016) and Zheng et al. (2024b) examined ageing’s effects on consumer behaviour more broadly, they did not address insurance as a distinct product category. Similarly, Guido et al. (2020) focused on financial asset management among the elderly, and Hettich et al. (2018) investigated older consumers’ decision-making in retail contexts, both overlooking insurance-related choices. Other studies, such as Yagi et al. (2022), explored health insurance literacy but did not examine purchasing intentions. Zheng et al. (2024a) conducted a scoping review on ageing’s impact on insurance purchasing intentions, providing valuable insights into general trends. However, their approach lacked a structured analytical framework, limiting the depth of theoretical, contextual, and methodological insights. Some reviews, such as Lambregts and Schut (2019), focus on specific insurance products like long-term care insurance, offering a narrow perspective that does not encompass broader ageing-related financial behaviours. Additionally, existing reviews predominantly centre on elderly consumers, overlooking how societal ageing influences the insurance behaviours of non-elderly populations. This gap highlights the need for a review that synthesizes these strands within a unified analytical structure and explicitly links ageing to insurance-related decisions.

This study addresses these gaps by systematically analysing how societal ageing shapes insurance purchasing intentions across different demographic groups. By employing the TCCM framework (theories-contexts-characteristics-methods; Paul & Rosado-Serrano, 2019), this research provides a structured evaluation of theoretical applications, contextual factors, consumer characteristics, and methodological approaches in this domain. Expanding the scope beyond the elderly, this study recognises ageing as a societal phenomenon that influences financial decision-making across life stages, offering a more comprehensive perspective on consumer behaviour in ageing societies.

The primary contribution of this study lies in its framework-based systematic review of how ageing influences insurance purchasing intentions. By introducing ageing risks (AR) as an explanatory construct, this study provides a structured approach to understanding how individuals perceive and respond to future financial and health-related uncertainties associated with societal ageing. Unlike prior reviews that focus predominantly on the physiological and psychological effects of ageing on the elderly, this study broadens the analytical scope to include non-elderly populations, recognising ageing as a societal phenomenon that shapes financial behaviour across life stages. Furthermore, by applying the TCCM framework, this study offers a structured evaluation of theoretical applications, contextual influences, consumer characteristics, and methodological approaches in this domain. Finally, by incorporating AR into an illustrative conceptual framework based on the Theory of Planned Behaviour (TPB), this study demonstrates how ageing-related perceptions can be integrated into established consumer behaviour models, providing a foundation for future empirical research.

The following sections detail the methodology, present results through the TCCM framework, discuss findings, propose conceptual framework, and recommend for future research. The conclusion synthesises key insights and outlines the study’s academic and practical implications.

## 2. Methodology

This systematic review follows the procedures established by the Preferred Reporting Items for Systematic Reviews and Meta-Analyses (PRISMA; see checklist in the Supplementary File) (Page et al., 2021). The TCCM framework was selected for its ability to systematically organise and synthesise theoretical approaches, data sources, contexts, and methodologies across diverse fields, facilitating a thorough and structured analysis (Paul & Rosado-Serrano, 2019; Paul et al., 2023). The methodology consists of the following three key stages: search strategy, study selection, and data extraction and synthesis.

### 2.1. Search Strategy

The search was conducted in September 2023, covering the following four major online databases: Web of Science, Scopus, ScienceDirect, and Emerald Insight. Four keyword sets were utilised to ensure comprehensive results. “Ageing” was included due to its relevance to both ageing population and its impact. Keywords such as “insurance,” “purchase,” and “intention,” along with their synonyms and related phrases, were used to capture studies on insurance purchasing intentions. These terms reflect widely adopted terminology in ageing and insurance research, and their selection aligns with keyword structures employed in prior reviews in this field (e.g., Zheng et al., 2024a). No restrictions, such as publication year or language, were applied due to the limited number of relevant studies. This approach aimed to comprehensively reflect the current scholarly landscape by minimising unnecessary screening criteria. Table 1 outlines the detailed search strategies and outcomes, with adjustments made using the advanced search functionalities of the respective databases.

A supplementary top-up search using the same databases and keyword sets was conducted in November 2025 to capture newly published studies. This update adhered to the original protocol and ensured that the evidence base reflected the most recent literature at the time of revision.

### 2.2. Study Selection

The study selection process involved four authors to minimise bias—two independently screened studies based on predefined criteria, with the remaining two reviewing the outcomes for consistency. Any disagreements were resolved through consensus. The inclusion criteria focused on empirical studies addressing both “ageing” and “insurance purchase intentions” within business disciplines such as management, marketing, and finance. Eligible studies were required to use micro-level data, focus on individual consumers, and be published in SCIE-/SSCI-indexed journals or ESCI-indexed journals with an impact factor (IF) greater than 1, following Basu et al. (2022) and Paul et al. (2023). Studies were excluded if they were non-empirical, relied solely on macro-level data, lacked a direct connection between ageing and insurance purchase intentions, originated from non-business fields (e.g., medical or biological disciplines), were published in journals not meeting the indexing or IF requirements, or were non-research formats (e.g., conference abstracts, book chapters). Articles for which full texts were unavailable were also excluded. An exception was made for Yeung et al. (2013), which, despite being a conference paper, was included for providing valuable academic insights relevant to this topic.

**Figure 1:**
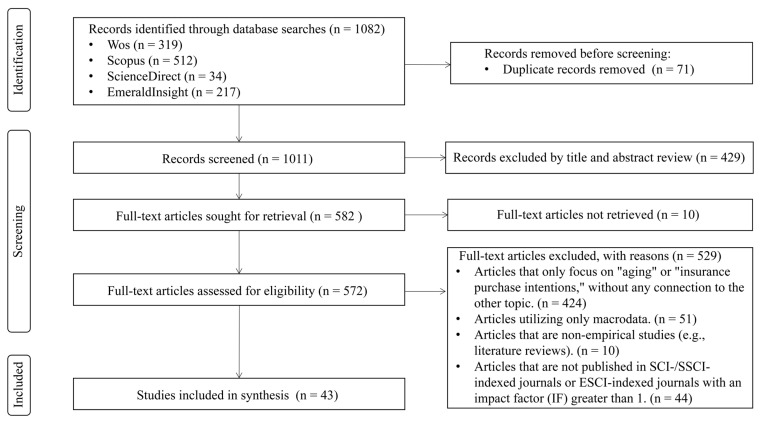
PRISMA flow diagram of studies search and selection.

The selection process is depicted in Figure 1. The search initially identified 1082 results across the four databases. After deduplication using EndNote 20, 1011 articles remained. A manual screening of titles and abstracts excluded 429 articles that did not align with the study’s objectives. Excluded articles primarily addressed medical topics such as disease treatment and healthcare resource allocation or focused on gerontology economics without relevance to micro-level insurance behaviour. This high exclusion rate is typical given the interdisciplinary and emerging nature of the research theme. Ultimately, 582 articles proceeded to full-text screening. Of these, 424 addressed only “ageing” or “insurance purchase intentions” without connecting the two; 44 were excluded for not being published in SCIE/SSCI-indexed journals or ESCI-indexed journals with an impact factor (IF) greater than 1; 51 relied on macro-level data; 10 were systematic reviews; and 10 were excluded due to inaccessible full texts. A total of 43 articles were synthesised for this review.

### 2.3. Data Extraction and Synthesis

The TCCM framework was applied during data extraction to systematically organise information, including general details (authors, title, publication year, journal), theories, contexts (countries and insurance types), characteristics (ageing-related antecedents, subject demographics, and decision variables), and methods (research design, data collection, sample size, and analysis techniques). Data extraction was conducted independently by two authors and cross-validated by the other two to minimise errors (Charrois, 2015; Gomersall et al., 2015). Given the inclusion of qualitative, quantitative, and mixed-methods research, a qualitative synthesis approach was used for analysis (Shaffril et al., 2021). Ethical approval and individual consent were deemed unnecessary as the review relied solely on publicly available data and fell within the exempt category for such requirements.

The core TCCM-based synthesis was undertaken using the 43 studies identified through the original search, whereas the 14 studies (Appendix A) located in the supplementary top-up search were incorporated as updated evidence without altering the primary dataset.

## 3. Results Based on TCCM Framework

Table 2 provides an overview of the results based on the TCCM framework (Paul & Rosado-Serrano, 2019). A detailed explanation follows.

### 3.1. Theories

Of the 43 articles analysed (Table 2), nearly half (20) did not specify the theoretical foundations of their investigations. Among those that did, the use of theories was highly varied. The most frequently employed is the Theory of Planned Behaviour (TPB), cited in 4 articles, followed by Utility Maximisation Theory in 3 articles. Theories such as Intrafamily Moral Hazard, Bounded Rationality, Insurance Demand, Rational Behaviour (TRA), and Prospect Theory are each mentioned in two articles. The remaining thirteen theories appear in only one article each.

### 3.2. Contexts

Table 2 summarises the geographical distribution and types of insurance covered in the analysed studies. The USA (13 articles) and China (12 articles) dominate the research focus on ageing and insurance purchase intentions. Europe, Spain, Australia, and the UAE each contributed two articles, while single articles from other countries reflect limited but notable geographic diversity.

Health insurance and long-term care insurance are the most studied types, appearing in 13 articles each. Life insurance and medical insurance follow with 5 articles each, while Islamic insurance (Takaful) and longevity annuities are examined in 3 articles each. Travel insurance is covered in 2 articles, and social old-age insurance is the least represented, with 1 article. This distribution underscores the prominence of health and long-term care insurance in ageing contexts, while also highlighting broader interest in various aspects of insurance purchase behaviour among older consumers.

### 3.3. Characteristics

#### 3.3.1. Subject’s Age

Of the 43 articles analysed (Table 2), 16 either do not specify age ranges or include overly broad categories, such as studies covering all age groups. Among the remaining 27 articles, 19 rely on primary data, with 11 focusing on elderly populations, and 8 addressing non-elderly groups. However, only 2 of the latter—Nosi et al. (2014) on young adults’ longevity annuities (aged 25–35) and Tam et al. (2021) on private health insurance among young Australians (18–30)—specifically target defined non-elderly demographics. The other six articles, such as Aziz et al. (2019) and Dragos et al. (2020), include non-elderly age ranges but focus on general factors influencing insurance purchase intentions rather than the specific impacts of ageing on this population. In contrast, studies on elderly populations demonstrate more targeted approaches, addressing their unique challenges and behaviours. This highlights a predominant focus on elderly groups, with limited research examining non-elderly populations in the context of ageing. Addressing this gap is essential for a comprehensive understanding of ageing’s societal impacts on insurance behaviours.

#### 3.3.2. Antecedents Related to Ageing

The selected articles, all related to ageing, either focus on insurance or specific populations and employ variables reflecting the impact of ageing (i.e., ageing-related antecedents). Table 2 lists eighteen such variables. Demographic factors are the most frequently used, including age (43 articles), marital status (23 articles), health status (22 articles), and the number of dependents (16 articles). These fundamental variables are crucial for understanding the ageing phenomenon and its implications for insurance purchase intentions. However, relying solely on demographic factors fails to fully capture the societal dimensions of ageing, highlighting the need for additional variables. Cognitive-related variables (e.g., cognitive abilities and age) are excluded for similar reasons.

Family-related variables, such as interpersonal influence (5 articles), caregiving experience (2 articles), and bequest motive (2 articles), emphasise the familial context’s role in shaping insurance decisions. Experiences like caregiving and family involvement significantly impact perceptions and decision-making regarding insurance. Risk-related variables are also prominent, with risk perception appearing in 7 articles and risk propensity in 3 articles, reflecting how individuals perceive and manage risks—an essential aspect of insurance decision-making.

Expectation-related variables, including family insurance expectations (self-insurance) (5 articles), anticipated dependence (4 articles), life expectancy expectations (2 articles), public insurance expectations (2 articles), and perceived economic instability (1 article), capture forward-looking considerations. These variables highlight the anticipatory nature of ageing and its influence on insurance purchase intentions, offering deeper insights into how individuals plan for future uncertainties.

#### 3.3.3. Corresponding Decision

The corresponding decision variables shed light on insurance-related behaviours and outcomes in the context of ageing. Purchase intention is the most frequently studied variable (21 articles), followed by insurance purchase status (9 articles), willingness to pay (9 articles), decision-making performance (3 articles), purchase preference (3 articles), frequency of health insurance use (1 article), and insurance purchase priority (1 article). These variables are essential for understanding individuals’ decision-making processes and outcomes in an ageing society.

### 3.4. Methods

The methodologies outlined in Table 2 reveal several key trends. Quantitative methods dominate, employed in 38 articles, while only 3 use qualitative methods and 2 adopt mixed methods, reflecting a strong reliance on statistical data to study insurance purchase intentions in the context of ageing.

Data collection is conducted primarily through questionnaires (23 articles) and secondary data sources (17 articles), with focus groups and experiments used less frequently (3 and 2 articles, respectively), offering additional qualitative and controlled insights. Sample sizes vary widely, with 12 articles using large national databases, 2 drawing from international databases, and 2 relying on local insurer databases. Primary data studies range from 23 participants in qualitative research to 7480 respondents in quantitative surveys, reflecting diverse research scales and contexts.

Regression analysis is the most common data analysis method, used in twenty-nine articles, with logistic, multinomial logit, and linear probability regressions being the primary techniques. Structural equation modelling (SEM), including partial least squares SEM (PLS-SEM), appears in six articles, addressing complex relationships and latent variables. The Contingent Valuation Method (CVM) is also employed in six articles to assess willingness to pay for insurance. Other methods are used in no more than three articles.

### 3.5. Updated Evidence from the Top-Up Search

The supplementary search conducted in November 2025 resulted in fourteen additional studies that met the predefined inclusion criteria. These studies reinforce and extend the patterns identified in the original synthesis. Several newly published papers continue to highlight the centrality of long-term care insurance in ageing societies, showing that subjective life expectancy influences life insurance uptake (X. Chen et al., 2024), cognitive abilities shape long-term care insurance demand (Gousia, 2023), and information interventions can strengthen long-term care insurance intentions among older adults (Liu et al., 2025). Evidence from discrete choice experiments further clarifies preference structures among older populations (Jin et al., 2024), while research on mental health spillovers highlights broader behavioural consequences of long-term care insurance participation (Y. Chen & Zhao, 2023).

Family roles and intergenerational expectations also emerge strongly in recent work. Studies show that children’s cohabitation and family asset reserves affect participation in commercial pension insurance (Feng et al., 2025), that pension responsibility norms and financial literacy shape willingness to purchase pension products (Han et al., 2025), and that children continue to function as a form of informal insurance shaping private insurance adoption among older adults (Ci, 2025). Additional evidence demonstrates that media exposure can moderate socio-economic inequalities in health insurance enrolment among older individuals (Barman et al., 2025) and that perceptions of pension responsibility influence pension insurance choices (Zhou et al., 2025).

Recent studies also expand attention beyond older adults. Research with young adults shows that loss aversion and temporal orientation influence life insurance decisions (Blanchard & Trudel, 2024), while work on participation in individual pension systems provides further evidence on behavioural drivers among working-age populations (Long et al., 2024). Contextual and structural influences are highlighted in investigations of implementation barriers in endowment insurance programmes within occupational settings (Cheng et al., 2023). Importantly, a recent cross-cultural study integrates ageing risks with behavioural theories to explain insurance purchase intentions across countries, offering conceptual alignment with the present review (Zheng et al., 2025b).

Taken together, the new evidence corroborates the core findings of this review: ageing-related expectations, intergenerational responsibilities, health and longevity perceptions, and socio-economic factors remain central determinants of insurance behaviours across demographic groups. While these studies broaden geographical and methodological coverage, they do not alter the main conclusions.

## 4. Discussion of Key Aspects

### 4.1. Lack of Direct Integration of Ageing into Insurance Research

Concerning the research content, scholars have extensively discussed specific factors influencing insurance purchasing intentions, such as cost, trust, brand equity, peer effects, and financial literacy (Reid et al., 2016; H. Lin & Prince, 2016; Rizwan et al., 2021; Kai et al., 2021; D. Li et al., 2022). However, these discussions rarely incorporate ageing as a factor or explore variables that explicitly reveal its impact on purchase intentions. Although certain variables, such as bequest motives, anticipated dependence, and risk perception (H. Lin & Prince, 2016; Xu et al., 2020; Choe et al., 2022), may partially reflect the influence of ageing, they are not intentionally linked to the ageing phenomenon. Similarly, studies focusing on specific types of insurance or populations often limit their scope to insurance products for ageing or the elderly (e.g., Guillemette et al., 2016; Kai et al., 2021). There is a notable lack of comprehensive research addressing the societal impacts of ageing on insurance purchasing intentions. In demand estimation studies, ageing is rarely considered unless the focus is directly on ageing-related insurance or populations. These studies primarily aim to determine pricing strategies to enhance consumers’ willingness to pay insurance premiums (e.g., Ying et al., 2007; Sowa et al., 2018). Overall, most existing studies only partially address the relationship between ageing and insurance purchasing intentions, leaving this topic largely unexplored.

### 4.2. Overemphasis on Elderly Consumers While Neglecting Non-Elderly Populations

The analysis of population age characteristics reveals that current studies predominantly focus on the elderly, with limited attention given to non-elderly individuals. This aligns with the preceding discussion, highlighting a fragmented exploration of ageing’s impact on consumer behaviour. For instance, studies often focus on specific types of insurance or ageing-related populations, neglecting the broader societal effects of ageing. Ageing, as a societal phenomenon, extends its influence across all age groups. Apart from directly affecting the elderly, it also impacts non-elderly individuals, such as adult children, younger generations, and caregivers (Pinquart & Sörensen, 2003; Zheng et al., 2024b). Among the reviewed studies, only Nosi et al. (2014) and Tam et al. (2021) specifically examined non-elderly individuals’ insurance purchase intentions. However, these studies merely analysed influencing factors without linking them to the ageing phenomenon. This underscores a significant gap in understanding how ageing affects insurance purchasing intentions among non-elderly populations.

### 4.3. Fragmented Use of Ageing-Related Variables Without Conceptual Clarity

Another key aspect involves the manifestations of ageing’s impact. As indicated in the results, several variables—such as risk perception, interpersonal influence, family insurance expectations (self-insurance), saving behaviour, anticipated dependence, and risk propensity—are frequently used to illustrate these impacts. Among these, variables related to future expectations (e.g., family insurance expectations and anticipated dependence) effectively reflect the influence of ageing, particularly on non-elderly individuals whose outlooks are intricately shaped by ageing-related considerations. Variables such as risk perception and risk propensity capture consumers’ attitudes towards risks associated with ageing, while interpersonal influence highlights the social effects of ageing, akin to subjective norms in the TPB (Ajzen, 1985, 1991). Saving behaviour relates to perceived behavioural control in the TPB, indicating whether individuals have sufficient resources for insurance decisions. As TPB is the most frequently employed theory in this field, future research should incorporate its key components—attitude, subjective norms, and perceived behavioural control—while integrating variables related to risk and expectations to provide a more comprehensive understanding of ageing’s impact on insurance purchasing intentions.

Furthermore, the variables identified in this study are synthesised from existing research. There may be additional variables yet to be employed that are equally relevant for reflecting the effects of ageing, representing a potential direction for future research. This indicates an opportunity to explore latent research gaps and expand the understanding of ageing’s broader societal impacts.

## 5. Conceptual Framework: The Role of Societal Ageing in Insurance Purchase Intentions

The findings from this review highlight critical gaps in the literature regarding the role of ageing in insurance decision-making. While existing research acknowledges variables such as risk perception, anticipated dependence, and family insurance expectations, these constructs are rarely conceptualised within the broader context of societal ageing. Furthermore, the predominant focus on elderly consumers has led to an overlooked yet significant research gap concerning non-elderly individuals and their insurance-related behaviours in response to demographic ageing trends.

To address this gap, this study proposes ageing risks (AR) as an explanatory construct that captures the perceived future financial, health, and intergenerational implications of demographic ageing. While previous research has examined ageing-related factors in isolation, these elements have not been systematically consolidated into a structured concept that reflects how individuals interpret and respond to societal ageing.

To illustrate its application, this study employs the Theory of Planned Behaviour (TPB) (Ajzen, 1991) as a demonstrative framework. TPB has been widely applied in insurance research (Dragos et al., 2020; Rizwan et al., 2021) and provides a structured approach to examining how ageing risks shape key determinants of decision-making. While TPB is used here as an illustrative model, the conceptualisation of AR is not restricted to this framework and can be extended to other behavioural theories in future research.

The proposed framework conceptualises AR as an independent variable representing individuals’ perceived exposure to future financial and health-related uncertainties associated with societal ageing, making it particularly suitable for examining the impact of societal ageing on non-elderly individuals. Unlike traditional ageing-related constructs, which predominantly focus on the elderly, AR captures how demographic ageing shapes the expectations, perceived risks, and financial behaviours of younger populations, including working-age adults and those with ageing family members. By incorporating AR into behavioural models, this framework enables a more comprehensive analysis of how non-elderly individuals anticipate and respond to the long-term economic and caregiving implications of an ageing society, influencing their insurance-related decisions. The model hypothesises that AR shapes the key components of TPB in the following ways.

First, AR shapes individuals’ attitudes towards insurance. Individuals who perceive greater ageing risks such as rising healthcare costs, pension inadequacy, and economic uncertainty in later life are more likely to develop a favourable attitude towards insurance as a risk-mitigation tool (Costa-Font & Courbage, 2015; Yeh et al., 2021). Studies have shown that heightened awareness of longevity risks increases the perceived necessity of insurance, particularly among non-elderly individuals planning for later life (He et al., 2023).

Second, AR influences subjective norms. AR reinforces social and familial expectations regarding financial preparedness for ageing-related risks. Individuals embedded in cultures that prioritise intergenerational financial responsibility may experience greater social pressure to secure insurance, particularly from family members (Tennyson & Yang, 2014; Broyles et al., 2016). In collectivist societies, interpersonal influence plays a key role in shaping perceptions of ageing risks, further amplifying the perceived social obligation to purchase insurance (Kai et al., 2021; Wu & Gong, 2023).

Third, perceived behavioural control is shaped by AR. AR interacts with economic constraints and policy uncertainty, shaping an individual’s perceived ability to act upon insurance intentions. If ageing risks are perceived as manageable through insurance coverage, individuals may exhibit greater confidence in their purchasing decisions. Conversely, if the financial burden of ageing is perceived as insurmountable, perceived behavioural control may decline, deterring insurance uptake (Costa-Font & Rovira-Forns, 2008; Wang et al., 2021a).

**Figure 2:**
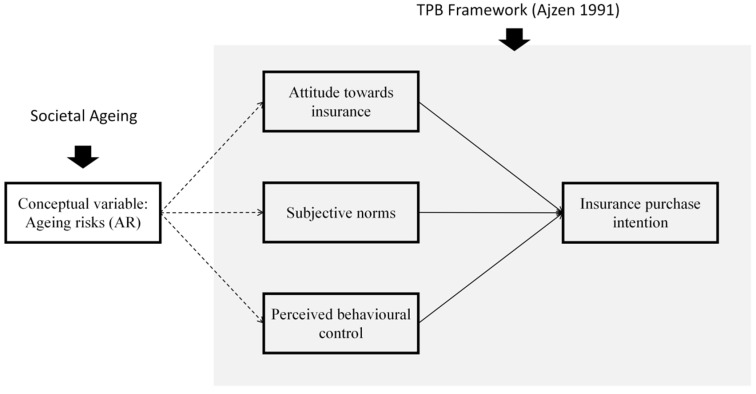
Proposed conceptual framework, adapted from Ajzen’s (1991) Theory of Planned Behavior.

By positioning AR as an independent variable, this framework provides a structured means to analyse how societal ageing translates into individual insurance purchase intention. While TPB is employed to demonstrate its applicability, the conceptualisation of AR is not limited to this model and can inform broader theoretical discussions on consumer decision-making and financial behaviour. Figure 2 presents the proposed framework.

## 6. Future Research Directions

The findings of this study highlight several gaps in the literature and suggest opportunities for future research to deepen understanding of how ageing impacts insurance purchasing intentions.

First, future research should empirically validate and refine ageing risks (AR) as an explanatory construct, ensuring its theoretical and methodological robustness. While this study conceptualises AR as an independent variable capturing individuals’ perceptions of financial and health-related uncertainties associated with societal ageing, further research is needed to standardise its measurement. Developing an “ageing risks index” would enable systematic assessment of how ageing perceptions influence financial decision-making, facilitating empirical analysis across different socioeconomic and cultural contexts. Longitudinal studies could track changes in individuals’ perceived ageing risks over time, providing insights into their evolving impact on insurance behaviour. Additionally, testing AR within alternative theoretical frameworks—such as the Health Belief Model (to explore its role in risk-prevention behaviour), Prospect Theory (to examine how AR shapes insurance-related risk framing), or Self-Determination Theory (to assess its interaction with financial autonomy)—would enhance its applicability beyond TPB.

Second, expanding geographical and product scope remains a critical research priority. Existing studies are predominantly concentrated in the USA and China, limiting the generalisability of findings to diverse socioeconomic and regulatory contexts. Future research should examine how variations in pension systems, public healthcare coverage, and cultural attitudes towards ageing shape insurance purchasing behaviour in underrepresented regions, particularly in emerging markets. Additionally, while much of the literature focuses on health and long-term care insurance, examining the impact of ageing risks on other insurance types, such as life, household, or travel insurance, could provide a more comprehensive perspective on consumer financial protection strategies.

Third, research should move beyond the elderly and explore how non-elderly populations respond to societal ageing. Ageing is a demographic shift that influences financial planning across all age groups, particularly working-age individuals and those with ageing relatives. Future studies should investigate how younger adults, particularly those anticipating caregiving responsibilities, adjust their insurance decisions in response to perceived ageing risks. Understanding these indirect effects is crucial for developing age-inclusive insurance policies that address long-term financial security concerns beyond the elderly population.

Finally, future research should integrate qualitative and mixed-methods approaches to complement predominantly quantitative studies. While quantitative models provide statistical validation, they often fail to capture the nuanced decision-making processes underlying insurance behaviour. In-depth qualitative studies, such as interviews and case studies, could offer richer insights into how personal experiences with ageing influence financial planning and insurance uptake. Combining qualitative insights with quantitative modelling would provide a more holistic understanding of consumer responses to societal ageing.

In summary, future research should focus on validating and extending the AR construct, broadening geographical and insurance product diversity, examining non-elderly populations, and incorporating mixed-methods approaches. Addressing these gaps will advance theoretical and empirical understanding of how societal ageing shapes insurance purchasing intentions. Table 3 provides specific research questions corresponding to these future research directions.

## 7. Conclusions

This study systematically reviewed the literature on the impact of ageing on individual consumers’ insurance purchasing intentions, following the PRISMA guidelines (Page et al., 2021) and employing the TCCM framework (Paul & Rosado-Serrano, 2019). A rigorous screening process identified 43 relevant articles from an initial pool of 1082. The analysis revealed significant gaps, including a limited focus on non-elderly populations, insufficient exploration of ageing-specific variables, and a concentration on particular geographical regions and insurance types. To address these gaps, this study introduces ageing risks (AR) as an explanatory construct that captures individuals’ perceived financial and health-related uncertainties associated with societal ageing. By integrating AR into a structured conceptual framework, exemplified through TPB, this study provides a foundation for future empirical research on how ageing perceptions shape insurance purchasing decisions.

The theoretical contribution of this study lies in highlighting the fragmented use of theoretical frameworks, particularly the lack of explicit ageing-related variables within established models such as the Theory of Planned Behaviour (TPB). By conceptualising AR as a structured explanatory variable, this study offers a systematic approach to incorporating ageing-related concerns into consumer behaviour research. While TPB serves as an illustrative example, AR can be adapted to other theoretical perspectives, broadening its relevance to financial decision-making in ageing societies.

From a practical perspective, this study underscores the importance of understanding how societal ageing influences insurance purchasing decisions across different populations and contexts. Incorporating AR into consumer segmentation strategies could help insurers develop products that address financial and caregiving uncertainties associated with ageing. Expanding insurance offerings beyond traditional health and long-term care policies to include products that mitigate broader ageing-related financial risks could improve accessibility and market penetration.

In conclusion, this study not only identifies critical gaps and emerging trends in the literature but also sets a research agenda for addressing the complexities of societal ageing. By conceptualising AR and outlining key research priorities—including its empirical validation, expansion across geographical and product contexts, and its impact on non-elderly populations—this study provides a structured foundation for advancing both theory and practice. As with prior reviews in this field, the synthesis is shaped by the heterogeneity of ageing-related measures across studies, which limits the degree to which constructs can be fully standardised. Moreover, the supplementary top-up search expanded the evidence base but was incorporated as updated contextual support rather than reanalysed within the core TCCM dataset to maintain methodological consistency. These limitations highlight opportunities for future research to advance more unified conceptualisations and broader empirical coverage, thereby supporting the sustainable development of insurance markets in ageing societies.

## Figures and Tables

**Table 1:** Search strategies used in the systematic literature review.

Database	Search Strategy	Search Results
Web of Science (WOS)	(((TS = (aging OR ageing OR senescence)) AND TS = (insurance)) AND TS = (purchas* OR buy* OR acquire OR obtain OR shopping OR procure OR decision-making)) AND TS = (intent* OR desire OR willingness OR motivation)	319
Scopus	(TITLE-ABS-KEY(aging OR ageing OR senescence) AND (insurance) AND (purchas* OR buy* OR acquire OR obtain OR shopping OR procure OR decision-making) AND (intent* OR desire OR willingness OR motivation))	512
ScienceDirect	(aging OR ageing) AND (insurance) AND (purchase OR buy OR decision-making) AND (motivation OR intention OR willingness)	34
Emerald Insight	abstract:“insurance” AND (abstract:“aging” OR “ageing” OR “senescence”) AND (abstract:“purchase” OR “purchasing” OR “buying” OR “buy” OR “obtain” OR “shopping” OR “acquire” OR “procure” OR “decision-making”) AND (abstract:“intention” OR “intent” OR “motivation” OR “willingness” OR “desire”) + title:“insurance” AND (title:“aging” OR “ageing” OR “senescence”) AND (title:“purchase” OR “purchasing” OR “buying” OR “buy” OR “obtain” OR “shopping” OR “acquire” OR “procure” OR “decision-making”) AND (title:“intention” OR “intent” OR “motivation” OR “willingness” OR “desire”)	217
Total	-	1082

The asterisk (*) functions as a truncation operator that retrieves all word variants sharing the same root.

**Table 2:** Comprehensive overview of the results based on TCCM framework.

Article	Theories	Contexts		Characteristics			Methods			
		Country	Insurance Types	Antecedents Related to Ageing	Subject’s Age	Corresponding Decision	Research Design	Data Collection	Sample Size	Data Analysis
Mellor (2001)	Theory of intrafamily moral hazard	USA	long-term care insurance	age; family insurance expectations (self-insurance); marital status; health status	50+	whether purchased insurance; purchase intention	quantitative	secondary data	Large national database	single-equation probit regression; bivariate probit regression; ordered probit regression
Dong et al. (2003)	not specified	Burkina Faso	health insurance	age; marital status	20–70	willingness to pay	quantitative	questionnaire survey	800 families	contingent valuation method (take-it-or-leave-it (TIOLI); bidding game)
Hanoch and Rice (2006)	Theory of bounded rationality	USA	health insurance	age; cognitive abilities	65+	decision-making performance	qualitative	secondary data	/	content analysis
Taylor and Ward (2006)	not specified	UK	medical insurance	age; health status; marital status; number of dependents; saving behaviour	not specified	whether purchased insurance	quantitative	secondary data	Large national database	random-effects logistic regression
Löckenhoff and Carstensen (2007)	Theory of positivity effect	USA	medical insurance	age; health status	22–39; 62–93	decision-making performance	quantitative	questionnaire survey	120	correlation analysis
Ying et al. (2007)	not specified	China	health insurance	age; marital status; health status	not specified	willingness to pay	quantitative	secondary data	Large national database	logistic regression
Costa-Font and Rovira-Forns (2008)	not specified	Spain	long-term care insurance	age; risk perception; family insurance expectations (self-insurance); health status; number of dependents	55+	willingness to pay	mix	questionnaire survey + focus group	400	contingent valuation method (single-bounded discrete referendum); probit regression
Costa-Font and Font (2009)	Utility maximisation theory	Spain	long-term care insurance	age; health status; risk perception	55+	willingness to pay	mix	questionnaire survey + focus group	400	discrete choice experiment (DCE); linearized logistic regression
Rice et al. (2010)	Theory of bounded rationality	USA	health insurance	age; health status	65+	purchase preference	quantitative	secondary data	Large national database	weighted logistic regression; weighted multinomial regression
Szrek and Bundorf (2011)	not specified	USA	health insurance	age; cognitive abilities	65+	purchase intention	qualitative	experiment	281	logistic regression
Shafie and Hassali (2013)	not specified	Malaysia	health insurance	age; health status; presence of private insurance coverage; marital status	not specified	purchase preference; willingness to pay	quantitative	questionnaire survey	472	contingent valuation method (bidding game); multinomial logit regression
Yeung et al. (2013)	not specified	China (HK)	life insurance, health insurance	age; health status; marital status; number of dependents	not specified	whether purchased insurance	quantitative	questionnaire survey	/	logistic regression
Nosi et al. (2014)	Theory of Reasoned Action (TRA)	Italy	longevity annuity insurance	age; interpersonal influence	25–35	purchase intention	quantitative	questionnaire survey	7480	PLS-SEM
Tennyson and Yang (2014)	Rational economic theory	USA	long-term care insurance	age; caregiving experience; health status; marital status; number of dependents	50–72	purchase intention	quantitative	secondary data	Large national database	Two-limit Tobit model
Costa-Font and Courbage (2015)	State-dependent utility framework; Theory of intrafamily moral hazard	Europe	long-term care insurance	age; family insurance expectations (self-insurance); public insurance expectations; risk perceptions; life expectancy expectations; anticipated dependence; health status	not specified	purchase intention	quantitative	secondary data	Large international database	linear probability regression
Zhang (2015)	not specified	China	social old-age insurance	age; number of dependents; presence of private insurance coverage	50–64	purchase intention	quantitative	secondary data	Large national database	difference-in-differences (DID) analysis
Broyles et al. (2016)	Life course theory	USA	long-term care insurance	age; caregiving experience; interpersonal influence; perceived economic instability	28–73	purchase intention	qualitative	focus group	59	content analysis
Guillemette et al. (2016)	Utility maximisation theory	USA	longevity annuity insurance	age; health status; marital status; risk propensity	not specified	purchase intention	quantitative	secondary data	Large national database	linear probability regression
H. Lin and Prince (2016)	not specified	USA	long-term care insurance	age; bequest motive; health status; marital status; number of dependents	50–69	whether purchased insurance	quantitative	secondary data	Large national database	linear probability regression
Nosratnejad et al. (2016)	not specified	Iran	health insurance	age; marital status; number of dependents; public insurance expectations	not specified	whether purchased insurance	quantitative	secondary data	Large national database	logistic regression
Price et al. (2016)	not specified	USA	medical insurance	age; cognitive abilities; health status; marital status	65–80	decision-making performance	quantitative	experiment	23	repeated measures analysis
Reid et al. (2016)	not specified	USA	medical insurance	age	60+	purchase intention	quantitative	secondary data	Large national database	conditional logit regression
Ampaw et al. (2018)	Insurance demand theory	Ghana	life insurance	age; number of dependents; health status; marital status	not specified	whether purchased insurance	quantitative	secondary data	Large national database	logit regression
Sowa et al. (2018)	not specified	Australia	health insurance	age	not specified	willingness to pay	quantitative	secondary data	Large national database	microsimulation approach
Wang et al. (2018)	not specified	China	long-term care insurance	age; health status; marital status	not specified	willingness to pay	quantitative	questionnaire survey	1743 families	contingent valuation method (bidding game); random-effects logistic regression
Aziz et al. (2019)	Intention-behaviour theories (TPB, TRA); Commitment-trust theory	Pakistan	Islamic insurance (takaful)	age; marital status	24–50	purchase intention	quantitative	questionnaire survey	224	PLS-SEM
Paton Schmidt (2019)	Cue utilisation theory	USA	Islamic insurance (takaful)	age; cognitive abilities	average 38	purchase intention	quantitative	questionnaire survey	371	correlation analysis; linear regression
Tolani et al. (2019)	not specified	UAE	health insurance	age; risk perception; health status; marital status	not specified	willingness to pay	quantitative	secondary data	Local insurer database	double hurdle model; neural network
Dragos et al. (2020)	Theory of Planned Behaviour (TPB)	Romania	life insurance	age; marital status	18–65	whether purchased insurance; purchase intention	quantitative	questionnaire survey	1579	logit regression; multinomial logit regression
Ondruška et al. (2020)	not specified	Slovak	life insurance	age; marital status; number of dependents; saving behaviour	18–62	purchase intention	quantitative	questionnaire survey	870	binary logistic regression
Xu et al. (2020)	not specified	China	long-term care insurance	age; marital status; number of dependents; saving behaviour; interpersonal influence; anticipated dependence	45+	purchase intention	quantitative	questionnaire survey	3987	multivariate logistic regression
Colón-Morales et al. (2021)	not specified	USA	health insurance	age; marital status; number of dependents	not specified	frequency of using health insurance	quantitative	questionnaire survey	126	Spearman’s correlation test
Eling et al. (2021)	Prospect theory; Utility theory	Europe	life insurance, long-term care insurance	age; marital status; number of dependents; health status; life expectancy expectations; risk propensity; saving behaviour	50+	whether purchased insurance	quantitative	secondary data	Large international database	binary logit model
Rizwan et al. (2021)	Theory of Planned Behaviour (TPB)	UAE	Islamic insurance (takaful)	age	not specified	purchase intention	quantitative	questionnaire survey	300	SEM
Tam et al. (2021)	Situational Theory of Problem Solving; Theory of Planned Behaviour (TPB)	Australia	health insurance	age; health status	18–30	purchase intention	quantitative	questionnaire survey	594	SEM
Wang et al. (2021a)	Random Utility Theory	China	long-term care insurance	age; marital status; number of dependents; health status	20–75	purchase preference	quantitative	questionnaire survey	1067	discrete choice experiment (DCE)
Wang et al. (2021b)	Insurance demand theory	China	health insurance	age	not specified	priority of insurance purchase	quantitative	secondary data	Local insurer database	multinomial logit regression
Kai et al. (2021)	Theory of peer effects	China	travel insurance	age; cognitive age; risk perception; interpersonal influence	65+	purchase intention	quantitative	questionnaire survey	1023	logistic regression
Yeh et al. (2021)	Expected utility theory (EUT); Prospect theory	China (TW)	long-term care insurance	age; marital status; number of dependents; risk propensity; health status; family insurance expectations (self-insurance)	not specified	whether purchased insurance; purchase intention	quantitative	questionnaire survey	1373	multinomial logistic regression
Choe et al. (2022)	not specified	Korea	travel insurance	age; risk perception; number of dependents	not specified	willingness to pay	quantitative	questionnaire survey	470	contingent valuation method (double-bounded dichotomous choice)
D. Li et al. (2022)	Trust transfer theory	China	medical insurance	age	40+	purchase intention	quantitative	questionnaire survey	247	PLS-SEM
He et al. (2023)	not specified	China (HK)	long-term care insurance	age; bequest motive; cognitive abilities; anticipated dependence; health status; number of dependents; marital status; family insurance expectations (self-insurance)	50–59	purchase intention	quantitative	questionnaire survey	1105	discrete choice experiment (DCE)
Wu and Gong (2023)	FBM (Fuzzy Trace Theory); UTAUT (Unified Theory of Acceptance and Use of Technology)	China	longevity annuity insurance	age; interpersonal influence; anticipated dependence; risk perception	20–49	purchase intention	quantitative	questionnaire survey	462	SEM

**Table 3:** Overview of future research directions and questions.

Directions	Research Questions Examples
Validating and Extending the AR Construct	1. How can an ageing risks index be developed and validated to measure perceived financial and health uncertainties associated with societal ageing?
	2. How do different dimensions of ageing risks (e.g., financial security, healthcare affordability, intergenerational dependency) uniquely influence insurance purchase intentions?
	3. To what extent does exposure to ageing-related events (e.g., parental caregiving, retirement planning) influence individuals’ perceptions of ageing risks and subsequent insurance decisions?
Broadening Geographical and Insurance Product Diversity	4. How do pension system differences across countries shape consumers’ reliance on private insurance for ageing-related financial security?
	5. How do regulatory frameworks in emerging markets influence the accessibility and affordability of insurance products for ageing-related needs?
	6. What role does cultural variation in intergenerational financial responsibility play in shaping demand for long-term care and life insurance?
Examining Non-Elderly Populations	7. How do anticipated caregiving responsibilities influence young adults’ willingness to purchase long-term care or life insurance?
	8. To what extent do financial concerns about ageing parents’ medical expenses affect middle-aged individuals’ insurance decisions?
	9. How do workplace ageing policies and retirement expectations influence younger employees’ engagement with health and pension insurance?
Incorporating Mixed-Methods Approaches	10. How do qualitative interviews with family caregivers reveal decision-making patterns that differ from survey-based findings on ageing and insurance?
	11. How does experimental manipulation of risk communication about ageing-related financial insecurity affect insurance purchase intentions?
	12. What insights can be gained from longitudinal studies tracking individuals’ insurance decisions as they age and experience shifts in financial and caregiving responsibilities?

## Data Availability

Data sharing is not applicable to this review article since there was no generation or analysis of new data in the study.

## Supplementary Materials

The following supporting information can be downloaded at: https://www.mdpi.com/article/10.3390/bs16010143/s1.

## Appendix A. Updated Evidence from the November 2025 Top-Up Search

**No.**	**Article**
1	Chen, Y., & Zhao, H. (2023). Long-term care insurance, mental health of the elderly and its spillovers. Frontiers in public health, 11, 982656. https://doi.org/10.3389/fpubh.2023.982656
2	Cheng, B., Li, J., Han, Y., Zhang, T., Huang, J., & Chen, H. (2023). A qualitative investigation of barriers and facilitators involved in the implementation of endowment insurance in China’s construction industry. Buildings, 13(4), 1063. https://doi.org/10.3390/buildings13041063
3	Gousia, K. (2023). Cognitive abilities and long-term care insurance: Evidence from European data. The Geneva Papers on Risk and Insurance-Issues and Practice, 48(1), 68–101. https://doi.org/10.1057/s41288-021-00240-8
4	Blanchard, S. J., & Trudel, R. (2024). Life insurance, loss aversion, and temporal orientation: a field experiment and replication with young adults. Marketing Letters, 35(4), 575–587. https://doi.org/10.1007/s11002-023-09712-4
5	Chen, X., Guo, Y., Lu, C., Wang, Y., & Wen, H. (2024). Does subjective life expectancy matter in purchasing life insurance among middle-aged and older adult? Evidence from China. Frontiers in Public Health, 12, 1426366. https://doi.org/10.3389/fpubh.2024.1426366
6	Jin, W., Wang, J., & Hu, X. (2024). Preference of urban and rural older people in Shandong Province for long-term care insurance: based on discrete choice experiment. Frontiers in Public Health, 12, 1445273. https://doi.org/10.3389/fpubh.2024.1445273
7	Long, Z., Niu, J., & Yi, F. (2024). Driving factors and micro-mechanism of residents’ participation intention in individual pension system. Heliyon, 10(2). https://doi.org/10.1016/j.heliyon.2024.e24488
8	Barman, P., Karmakar, R., Roy, A., & Dakua, M. (2025). Mass Media Exposure Moderates the Association of Education and Wealth with Enrollment in Health Insurance Among Older Adults Aged 60 Years and Older in India. Journal of Aging & Social Policy, 37(3), 451–475. https://doi.org/10.1080/08959420.2024.2401713
9	Ci, Z. (2025). Children as insurance revisited: Impact of children on private insurance adoption among older parents. Journal of Risk and Insurance, 92(1), 116–138. https://doi.org/10.1111/jori.12492
10	Feng, C., Ying, Z., Yang, L., Wu, L., & Liu, B. (2025). How do children’s cohabitation and family asset reserves impact rural individuals’ participation in commercial pension insurance?. Frontiers in Public Health, 13, 1548241. https://doi.org/10.3389/fpubh.2025.1548241
11	Han, B., Liu, C., & Ling, W. (2025). Impact of financial literacy and awareness of children’s pension responsibilities on the willingness to purchase pension insurance: Evidence based on survey data (CLASS). Finance Research Letters, 76, 107015. https://doi.org/10.1016/j.frl.2025.107015
12	Liu, J., Hao, J., Maitland, E., Nicholas, S., Wang, J., & Leng, A. (2025). Shaping Long-term Care Insurance Intentions Among Chinese Adults Aged 50–70: Role of Information Interventions in Health Risks. Innovation in Aging, igaf054. https://doi.org/10.1093/geroni/igaf054
13	Zheng, Z., Hafizuddin-Syah, B. A. M., Zaki, H. O., & Tan, Q. L. (2025). From Aging Risks to Insurance Purchase Intentions: A Cross-Cultural Integration of Social Influence and Planned Behavior Theories. Journal of Consumer Behaviour. https://doi.org/10.1002/cb.70070
14	Zhou, L., Zhu, W., Cui, Y., Chen, Y., & Xu, X. (2025). Research on the impact of residents’ pension insurance choices based on their cognition of pension responsibility. Frontiers in Public Health, 13, 1592206. https://doi.org/10.3389/fpubh.2025.1592206
